# Barriers to quality of life in patients with multiple sclerosis: a qualitative study

**DOI:** 10.1186/s12883-022-02700-7

**Published:** 2022-05-13

**Authors:** Zahra Hosseini, Atefeh Homayuni, Masoud Etemadifar

**Affiliations:** 1 Health Education and Promotion, Social Determinants in Health Promotion Research Center, Hormozgan University of Medical Sciences, Bandar Abbas, Iran; 2 Student Research Committee, Hormozgan University of Medical Sciences, Bandar Abbas, Iran; 3 Neurology, Department of Neurosurgery, School of Medicine, Isfahan University of Medical Sciences, Isfahan, Iran

**Keywords:** Barriers, Content analysis, Multiple sclerosis, Qualitative research, Quality of life

## Abstract

**Background:**

Multiple sclerosis (MS) is a chronic progressive disease of the central nervous system that affects the patients’ quality of life. This research was conducted with the aim of identifying the barriers of quality of life in patients with MS.

**Methods:**

This qualitative study was conducted through a conventional content analysis approach. We used the purposeful sampling with maximum diversity in terms of gender, age, education, marital status and employment. Data were collected through semi-structured interviews with 18 patients with multiple sclerosis referred to the MS Association of Isfahan. Interviews were conducted to the point of information saturation.

**Results:**

Through the content analysis of the interviews, we identified 2 main categories and 11 sub-categories. The main categories include intrapersonal problems (physical problems, psychological disorders, turbulent future, functional limitations, job loss and pennilessness), and environmental barriers (disease and treatment process, fatigue of caregivers, information deficiency about MS, family tensions, lack of social support and fun and entertainment).

**Conclusions:**

In order to improve the quality of life in these patients, there is a need for attention and practical measures in the field of identified factors. By removing barriers such as providing educational and counseling services to the patients and their families, adapting the urban structure, providing financial support and adequate insurance coverage, the authorities can take measures to ensure patients’ health and improve their quality of life.

## Introduction

Multiple sclerosis (MS) is an autoimmune, inflammatory, chronic, and debilitating disease of the nervous system that is associated with various inflammatory manifestations, demyelination, and loss of axons [1]. There are 400,000 people with MS in the United States, and more than 2.5 million people worldwide live with the disease. The average age of diagnosis of MS is about 30 years, and MS is 2 to 4 times more common in women than men [2]. Common symptoms of MS range from anesthesia (paresthesia), weakness, visual disturbances, diplopia, imbalance, gait disorders, dizziness, spasm, ataxia, nystagmus, neuropathic pain, urinary urgency or retention, sexual dysfunction to depression, emotional-cognitive disorders and inability to tolerate heat [3].

This disease is one of the most debilitating diseases at a young age, which has created many challenges in terms of quality of life (QOL) for these patients. Patients with chronic and debilitating diseases such as MS face their disease-related problems [4]; these cause a comprehensive reduction of physical, social and cognitive functions [5] and ultimately affect negatively their QOL. Quality of life is a multidimensional concept. The World Health Organization defines it as an individuals’ perception of their position in life, in relation to the cultural context and value systems in which they live, as well as in relation to goals, expectations, their standards and concerns [6]. Low QOL can lead to inefficient coping and adjustment mechanisms and therefore increase stress and lead to increased disease severity [7].

In general, there are several factors that can significantly reduce or improve the QOL in people with MS. Studies show that the QOL in people with MS is predicted by physical factors including the severity and duration of the disease, weakness, disability, adaptation and gait disorders [8]. Areas related to psychological issues for the afflicted women and the areas of physical function and role limitation due to physical problems for the men have a greater impact on their QOL [9]. The results of some studies suggest that mood disorders, poor QOL and perceived fatigue [10], depression, anxiety, chronic fatigue, sleep problems, pain, sexual dysfunction [11] and sleep disorders, fatigue and depression [12] are among the common psychological problems in patients with MS; these affect the treatment process of these patients and reduce the patient’s active participation in the treatment process [13]. The results of Strober’s study on the patients with early-stage MS showed that patients with lower QOL scores reported more fatigue, sleep problems, pain, depression, and anxiety. They also reported lower levels of self-efficacy, control center, and social support. They showed higher levels of neuroticism, lower levels of extraversion, and higher levels of unwillingness to confront. Individuals with high QOL reported higher levels of general and MS-specific self-efficacy and internal locus of control and more perceived social support. These individuals were more likely to use problem-focused and adaptive coping strategies to cope with MS. The focus of control and anxiety were the most important predictors of QOL and explained 40% of the variance in QOL [14].

The development of an effective intervention program to improve the QOL in people with MS requires the identification of factors affecting the QOL in these patients, especially factors that inhibit their QOL. A review of studies showed that we have a huge amount of quantitative data on this topic, but quantitative research has intrinsic limitations in deepen the subjective experiences on a specific topic. In addition, conducting this research in the Iranian culture can help to clarify the nuances in socio-cultural factors affecting the QOL in these patients. Therefore, the present study was performed to identify the barriers of QOL in patients with MS.

## Methods

A qualitative methodology was selected to explore participants’ experiences and perspectives about barriers of QOL. This study was performed with the approach of conventional content analysis.

### Participants and recruitment

People with MS referred to the MS Association of Isfahan in 2019 participated in this study. Participants were purposefully selected based on theoretical sampling with maximum diversity (in terms of age, gender, education, marital status, employment status, etc.). Data collection continued until data saturation. First, the necessary permits were obtained. According to the previous coordination with the head of research affairs of the MS Association, the researcher attended the classes of the association and after establishing initial communication with patients, introduced herself and explained the purpose and importance of the research as well as the conditions for inclusion in the study. In order to participate in the study, a number of patients voluntarily announced their readiness. Finally, the necessary arrangements were made to interview those who were willing to participate in the study.

### Inclusion and exclusion criteria

In order to select the participants, the following inclusion criteria were used: 1) Patients with MS confirmed by a neurologist according to the 2017 McDonald criteria [15]. 2) Being passed at least 1 year since their diagnosis. 3) Having not a chronic disease other than MS. 4) Having rich and useful experiences about living with the disease and their desire to retell their experiences to the researcher. Exclusion criteria were: 1) being unable to cooperate and talk due to the worsening of the disease or other reasons. 2) Loss of any of the inclusion requirements during the interview.

### Ethical considerations

Prior to the interviews, all participants were given the information needed to participate in the interview. To ensure voluntary participation in the study, participants were asked to give their consent. All participants were given consent forms to sign. They were assured that the transcript of the interview would remain strictly confidential and that participants would not be named in the final description and analysis. The reason for using the voice recorder was also explained to them and the interviews were recorded with their permission.

### Data collection and analysis

The main tools for data collection were semi-structured in-depth interviews. Thus, 18 interviews were conducted (15 interviews with patients and 3 interviews with the patient’s relatives). The duration of each interview varied between 30–90 min. The location of the interviews was chosen by the participants themselves, which was at the place of residence or the MS Association. In the MS Association, the interview was conducted in a dedicated room while maintaining privacy and silence and creating the greatest comfort and satisfaction of the participant. At the beginning of each session, the interviewer asks the participants for demographic information, including age, age of disease onset, education level, marital status, and so on. Interviewees were free to cancel the interview whenever they wished. The interview was recorded by voice Recorder.

The interview questions began and continued with these questions:

1. How has this disease affected the various aspects of your life?
2. What are the most important factors that you think affect the quality of your life?
3. What factors are undermining your QOL?

Also, if necessary, we asked in-depth and exploratory questions to elaborate on the details, such as “Please explain more”, “Please give an example”, “If you have a memory about this, tell us”, “When you say … what do you mean?”, “How did you feel about this?”. During the interviews, the researcher monitored the participants’ reactions, feelings and emotions and, if necessary, took notes and, after rewriting the interviews, wrote these notes in the margins of the interview text. Interviews were first handwritten on paper and then typed.

Data were analyzed using Max-QDA version 10 software. After extracting the initial codes, items that were conceptually similar or related in meaning were placed in a category. This process of analysis continued until the emergence of the main and sub-categories.

### Methodological considerations

Acceptance, validity, confirmability and transferability criteria were used to determine the accuracy of the data [16]. In this research, the researcher first wrote her personal beliefs, values and judgments and how they make impact on data collection and analysis. She recorded her thoughts about the answers she expected to hear from the participants and tried to avoid emphasizing them during the research. In this study, we tried to increase the validity of the data by having enough presence in the research environment, by interviewing different participants and sharing the coding and text with the participants and the observers’ review. In the review by the participants, interpretations of some participants’ explanations were sent for review to see whether they are compatible with their experiences. Sampling with maximum diversity also contributed to the transferability and appropriateness of the data. In order to increase the data transferability, the researchers tried to provide a detailed, accurate and step-by-step description of how the research was conducted and the characteristics of the study population, to enable other researchers to follow the process.

## Results

61.1% of the participants were married and the majority of them had high school education and diplomas (38.9%). 72.2% of the participants were housewives. Participants ranged in age from 29 to 59 years with a mean age of 40.61 years old. The mean age of disease onset was 28.61 years old. Married patients participating in the study had a minimum of 1 and a maximum of 4 children. Four participants had a history of multiple sclerosis in their relatives (Table 1).

**Table 1:** Research participants’ demographic characteristics

Variable	Category	N (%)
Sex	Male	4(22.2)
	Female	14(77.8)
Marital status	Single	5(27.8)
	Married	11(61.1)
	Widowed	1(5.55)
	Divorced	1(5.55)
Education level	Elementary school	2(11.1)
	Junior high school	2(11.1)
	High school and diploma	7(38.9)
	Associate degree	3(16.7)
	Bachelor’s degree	4(22.2)
Employment	Housewife	13(72.2)
	Employed	3(16.7)
	Unemployed	2(11.1)
Frequency of hospitalization in the last 1 year	None	13(72.2)
	Once	4(22.2)
	Twice or more	1(5.6)

Two main categories and 11 sub-categories were obtained by data analysis: intrapersonal problems and environmental barriers. The extracted main categories and subcategories are shown in Table 2.

**Table 2:** Categories and sub-categories extracted from the analysis of patients' experiences

Category	Sub-category	Repetitive Codes
**Intrapersonal problems**	Physical problems	Sensory-motor problems, vision problems, speech and swallowing problems, cognitive disorders, bladder dysfunction, extreme fatigue, unstable body temperature, balance and gait disorders, impotence and sexual dysfunction, …
	Psychological disorders	Depression, stress, anxiety, bipolar disorder, personality disorders, loneliness, stressful thinking habits, emotional fatigue, …
	Turbulent future	Falling hopeless, worries about the future of the children, worries about saving life, worries about marriage and childbearing, …
	Functional limitations	Limitations in physical/ social and cognitive functions, physical dependence on others, …
	Job loss and pennilessness	Financial problems, leaving the job, forced to change job, losing job, disability and homelessness, …
**Environmental barriers**	Disease and treatment process	Disease progression, late diagnosis, rejection and incompatibility with disease, non-adherence to treatment, concealment of the disease by the patient, drug and therapeutic side effects, incompatibility with treatment, high cost of treatment, neglect of patients' rights by therapists, lack of easy access to medicine, lack of appropriate and sufficient empathy on the part of medical staff, …
	Fatigue of caregivers	Sense of helplessness, inability and despair in the caregiver, …
	Information deficiency about MS	Lack of knowledge or little knowledge of patients with MS and society about MS, the weakness of the media in portraying the disease, …
	Family tensions	Broken and unpleasant marital relationships, illness in the family, addiction of a family member, death of a loved one, divorce, conflicts within and between families, …
	Lack of social support	Compassionate attitudes, unwarranted sympathies, social misconceptions about MS, unwarranted jealousy, inappropriate family support for the patient, insufficient support of family and friends, …
	Fun and entertainment	Deprivation of presence in some places, …

In the following, we will describe each of the categories.

### Category 1: intrapersonal problems

This category includes five sub-categories: Physical Problems, Psychological Disorders, turbulent Future, Functional Limitations, and Job Loss and pennilessness.

#### Physical problems

MS can cause a wide range of physical problems, depending on which part of the central nervous system it affects. Most participants had sensory-motor problems, vision problems, speech and swallowing problems, cognitive disorders, bladder dysfunction, extreme fatigue, unstable body temperature, balance and gait disorders, impotence and sexual dysfunction, etc.:

“He has no balance, his left side is more involved; his left foot is paralyzed if he is tired, he has to pull it, he becomes numb, one has to hold his hand otherwise he will fall to the ground (mother of a 33-year-old patient)”.

#### Psychological disorders

Disorders such as depression, stress, anxiety, bipolar disorder, personality disorders, loneliness, stressful thinking habits, and emotional fatigue were among the most common psychological problems in participants. These problems were associated with the recurrence of symptoms and impaired patients’ social functioning:

“He was depressed because he could not work. Because he had no income and a sense of disability bothered him. His life became very involved. His relationships with his friends changed a lot (33-year-old patient’s sister).”

In some cases, the person is not able to perform many of their tasks as before and independently. This causes the patient to feel dissatisfied, inadequate with their abilities, and sometimes even to feel burden:

“I have a very bad feeling that my abilities have decreased. I am not satisfied with myself. For example, in the language class, the students realize that I am lacking. They learn a grammar with 1 time, teacher has to explain it 2-3 times for me. So I will be in trouble (29-year-old patient)”

#### Turbulent future

Fear of the future in relation to personal and social life issues was one of the things that deprived the patient and family members of their peace of mind by constantly engaging them. There were worries and concerns about marriage and childbearing, responsibilities and worries about the future of the children, and worries about saving life, and it bothered the person:

“My family, especially my parents, were more worried about my illness than I was. Because I had just had children. I had problems with my husband and his family before I got sick. My family was worried that this disease would affect the course of the problems (37-year-old patient)”.

The disease also caused patients to fall hopeless. The fall of dreams signifies the loss of the privileges of a normal life. Cases such as obstacles to starting a family, marriage due to compassion and not because of love and deprivation of the natural pleasures of life were among the cases that indicated the decline of people’s desires:

“My last semester of university, my illness started. Well, I could not get married. All my peers and classmates got married and went about their lives, their feelings, what about me? I was in the air (35-year old patient).”

#### Functional limitations

MS reduces a person’s physical, social and cognitive functions. MS can have a profound effect on everyday life, including leisure. Injuries caused by MS prevent the patient from effectively participating in leisure activities or people have to make changes in them to participate in these activities:

“I exercised a lot, I went mountaineering, I was a volleyball player. I can’t do these things now and this is the biggest torment for me. Why can’t I be naughty like then? (33-year-old patient)”

The disease also caused a change in the quality and quantity of patients’ social relationships. The participants’ experiences in this field showed that their participation in mourning ceremonies was limited, some of them were excluded by the society and their social and interpersonal interactions were reduced.

“I cannot be in a lot of groups of people, my friend wants to dance, smoke a hookah, drink alcohol and I cannot do any of these things. Many of them broke up with me. I cannot tell them I have this problem (29-year-old patient)”.

Patients have impaired physical activity and cannot easily perform their daily activities. These patients need the help of others in personal matters and home activities. On the other hand, some were never willing to seek help from others:

“He said I was sitting on the motorbike, I dropped my cell phone, I could not bend down and pick up my cell phone. “I told someone to come and get it, he thought I was joking or I wanted to tell him to do the same (mother of 35-year-old patient)”.

#### Job loss and pennilessness

Some participants stated that MS had disrupted their work and activities. Due to physical weakness, disability and sensory and cognitive disorders caused by the disease, patients have either lost their jobs and professions or have been forced to change jobs. Progression of the disease in some patients has even led to their disablement:

“After a few years of work experience, he had to change job because his job was such that he had to stand on his feet for a long time, and he needed a lot of attention and concentration. Well, he couldn’t. He fell short physically. He had to leave. He could not work at all for a while, he was unemployed (36-year-old patient’s wife).”

One of the problems that people often face after getting sick is the financial problems caused by the disease; it was raised by most of the participants. Among the problems that caused financial problems for patients were the inability to afford living expenses and the cost of a proper diet and financial dependence on others:

“My husband has a simple job. One day he has work, one day he does not have. With this situation, life is very difficult in general, so he is not able to pay for my medicine and my own expenses and the children’s (38-year old patient).”

### Category 2. Environmental barriers

This category refers to the barriers that arise from the environment, society, and relatives, and include six sub-categories: disease and treatment process, fatigue of caregivers, information deficiency about MS, family tensions, lack of social support and fun and entertainment.

#### Disease and treatment process

The results showed that some factors related to the disease such as disease progression, late diagnosis, rejection and incompatibility with disease, non-adherence to treatment and concealment of the disease by the patient are among the negative factors affecting QOL. Some patients, years after the onset of the disease, were still unable to accept and adapt to their disease. Some of them did not follow up to treat the disease, did not take their medications or took them irregularly. On the other hand, fear of judgment or trying to keep the family calm leads to hiding the disease and pretending to be healthy in some of them:

“Except for family members and one or two close friends, no one knows I’m sick. MS is settled down badly among people. Especially when I say I’m sick, they say that’s why I got divorced. “It didn’t matter. I filed for divorce myself (29-year-old patient).”

Most patients and their families are looking for a way to treat the disease after it has occurred, which may lead to difficulties and problems. Problems mentioned by the participants in this field include drug and therapeutic side effects, incompatibility with treatment, high cost of treatment, neglect of patients’ rights by therapists, lack of easy access to medicine (scarcity of foreign drugs due to sanctions and supply of medicine from special centers) and lack of appropriate and sufficient empathy on the part of medical staff:

“Night after night I was injected with fever and chills. During the night I could not sleep at all and in the morning I had no strength and energy at all. After a few months I was very upset. I said I would not take these ampules again. It was very hard. It was as if my life had been disrupted by this disease (40-year-old patient).”

#### Fatigue of caregivers

Every family member who takes care of a sick person is called a caregiver. The role of caregivers in the lives of patients, especially patients with disabilities, is important. Sometimes the lack of solution to problems or the existence of obstacles in solving problems, leads to a sense of helplessness, inability and despair in the caregiver; it was also mentioned by the participants in this study:

“They need a lot of care. Someone unemployed should care them, but I cannot, I’m all involved. I used to massage him, I used to lubricate his legs, now I cannot. I took him a bath, crying from the beginning to the end. I was alone, he is a man, heavy (patient’s 59-year-old wife).

#### Information deficiency about MS

Some participants complained about the inappropriate behavior of people in the society due to lack of information about MS and the weakness of the media in portraying the disease:

“Immediately after the affliction, I saw the film *Gold and Copper* (an Iranian film about MS). When I saw the film, I lost my spirit. The actor in the film had MS, she was disabled. Her husband did all the work. They were very miserable. That is, they made the disease look very bad (39-year-old patient).”

#### Family tensions

Most participants stated that traumatic family factors such as broken and unpleasant marital relationships, illness in the family, addiction of a family member, death of a loved one, divorce and conflicts within and between families are among the familial factors of the onset or exacerbation of the disease:

“Perhaps one of my biggest problems is that my illness was simultaneous with my mom’s cancer. My mom always takes care of me. I take care of her. If I get sicker, it’s all because of my mom’s illness. If there is an attack, I behave in a way that my mother does not notice (29-year-old patient).”

#### Lack of social support

One of the important factors affecting the QOL of these patients was insufficient support of family and friends. Some patients complained that family members did not accompany and understand or support them during their illness and treatment. Others said that family members’ speech and behavior made them feel powerless:

“My children expect me to be like before. They do not want to accept that I cannot. My daughter’s words sometimes bother me a lot, when she says you did not mother us (39-year-old patient).”

Some patients stated that the disease changed the family and society’s view of them emotionally. Compassionate attitudes, unwarranted sympathies, social misconceptions about MS, unwarranted jealousy, and inappropriate family support for the patient were among the inappropriate reactions that sometimes offended the patient and his or her family members:

“Do not feel sorry for me. I feel that these people are talking in a state of pity. It is as if I feel my heart is breaking. I say, I’m human, I’m living (40 -year-old patient).”

#### Fun and entertainment

Participants have also experienced deprivation of presence in some places. They believe that to solve this problem, sometimes the physical structure of public places in the city must be considered. They also point to the lack of support systems for proper planning, such as holding special sports and art classes for these people:

“When he went to the park with his friends, they would take him from his arms, to go up the stairs. These places must be fixed by the municipality. He must think of the person who has a wheelchair. Okay, but he does not (35-year-old sick mother).”

## Discussion

The aim of this study was to identify the barriers of QOL in patients with MS. The results of this research led to identifying 2 main categories (intrapersonal problems and environmental barriers) and 11 sub-categories. In the following, each of these categories and subcategories are discussed based on previous studies.

### Category 1: intrapersonal problems

#### Physical problems

Research results [17–19] show that many complications of MS have a negative effect on quality of life-related health factors. MS is a progressive disease of the central nervous system that causes sensory impairment, weakness, muscle cramps, visual impairment, cognitive impairment, fatigue, limb tremors, urinary incontinence, defecation disorders, sexual dysfunction, disorders of balance, forgetfulness, hearing loss, numbness, blurred vision, diplopia and speech disorders in the patient [20]. Together, these problems affect most of a person’s daily activities such as dressing, bathing, self-care, etc., and reduce personal independence, feelings of inadequacy, and also reduce a person’s QOL [21].

#### Psychological disorders

The results of the study showed that one of the factors affecting the QOL of patients was psychological disorders. This finding is consistent with the results of various studies [12, 22–25]. In this regard, a study showed that psychological disorders are the most effective factor in patients’ QOL [26]. The prevalence of psychological disorders in MS patients can be attributed to neurological factors, disease complications (pain and fatigue) and social factors. Fatigue is an inhibitory factor of effective activity that reduces patients’ QOL and makes them prone to psychological problems [23]. In contrast, the neuropsychological perspective attributes the prevalence of psychological symptoms to inflammation and destruction of the nerve sheath in people with MS [27]. Some studies also believe that the disease is associated with social isolation and disruption of social maps, and eventually psychological symptoms appear in response to a decrease in positive performance in people with MS [28].

#### Turbulent future

The results showed that most of the single subjects were concerned about the issue of marriage. These are concerns about being unable to start a family, the ability to have children, transmitting the disease through genetics to children, as well as not predicting the course of the disease and anxiety about the aggravation of symptoms during pregnancy. In line with the present study, Borisow et al. [29] emphasize that the issue of fertility and heredity in pregnancy in patients with MS is an issue that severely affects marital relationships in these individuals. On the other hand, the results showed that in the case of patients who experience stressful relationships and family conflicts, there is a concern that these relationships become severe due to the disease and the conflicts will take a more serious form; even in some cases, there are fears of separation and the breakdown of marital relationships. In this regard, the findings of a study conducted by Popp et al. [30] show that the disease does not lead to widespread family conflicts, but rather exacerbates existing family disputes and, in particular, existing conflicts between couples. Families with pre-illness cohesion may face challenges and differences due to illness, but this does not mean that these challenges will lead to the widespread conflicts.

#### Functional limitations

The results showed that the disease leads to a decrease in the level of activities of daily living and performing enjoyable activities. The onset of MS often causes an initial or complete reduction in a person’s physical, social, and cognitive functions and has a devastating effect on the QOL of the patient, family, and loved ones [31]. It can also have a profound effect on the person’s social activities. Functional changes such as limited mobility, premature fatigue, and problems with bowel and bladder control can make it impossible to continue participating in many social and occupational activities and will affect the patient’s relationships with others [32, 33]. This causes depression and isolation. The results also showed that these patients need the help of others in personal affairs and home activities. A study done by Huijbregts et al. [34] on the disability of 388 patients with MS showed that 71 patients (29%) were completely independent in their daily activities (bathing, dressing, combing their hair, and eating) and 86 patients (53%) needed help with their daily activities for at least one hour a day.

#### Job loss and pennilessness

MS is a chronic debilitating central nervous system disease that is associated with high unemployment rates in early adulthood [35, 36]. Many people with MS lose their jobs or have to change jobs due to symptoms such as fatigue, inability to function, and cognitive impairments. Hence, job may be considered as a sign of the patient’s overall performance, which has a significant impact on his QOL [37]. A research conducted by Lunde et al. [38] on 213 patients with MS in Western Norway showed that patients with relapsing–remitting MS and (RRMS) had higher employment rates than patients with primary progressive MS (PPMS) and secondary progressive MS (SPMS). Patients with higher education, lower age of onset, shorter duration of illness, less severe disability, and less fatigue were more likely to be employed. Among the problems and concerns of the participants in the research were the economic difficulties and the financial burden of the disease on the patient and the family. MS is associated with high direct and indirect costs. Direct costs include medical expenses such as hospitalization, inpatient care, and outpatient and pharmaceutical care. Indirect costs include costs associated with short-term and long-term disabilities, illness-related absences from work, workers’ compensation, and early retirement [39]. Having chronic diseases can deprive patients of comfort, limit treatment, and increase the severity of their problems by imposing staggering costs. Financial constraints are the most common barrier to accessing medical treatment for patients [40]. Chang et al. state that some factors such as economic problems, inability to earn money and financial support also affect people’s ability to adapt [41].

### Category 2: environmental barriers

#### Disease and treatment process

The results showed that some patients, despite the years after the onset of the disease, were still unable to accept their disease. Non-acceptance of the disease may lead to non-compliance with the disease and delay the treatment process [42]. Acceptance of the disease along with optimistic attitudes have been considered as strong predictors of QOL [43]. Another challenge and problem of the participants in this study was the problems related to the treatment process; not addressed in a timely manner, they may lead to discouragement of patients and their families and may prevent them from continuing the treatment process. The high cost of drug delivery, the cost of repeated laboratory tests, rehabilitation sessions, and the high cost of MRI had left most participants financially desperate. Most of the participants expressed dissatisfaction with the neglect of their rights by the medical staff and the superficial response to the patient and the lack of empathy and sufficient attention. We recommend that medical staff show the utmost empathy in responding to patients’ questions and feedback on their reactions and behaviors, which improves the relationships between patients and the treatment staff and leads to their satisfaction. Finally, another unbearable suffering of people with MS is the side effects of medications. Most participants acknowledge a decrease in ability and disruption in their daily activities due to the side effects of medication. The findings of Samkoff’s study also confirm the side effects of the drugs. According to his research, in the treatment of MS with all forms of interferon beta, side effects such as fever, chills, headache, etc. are observed, which manifests itself in three degrees: mild, moderate and severe [44].

#### Fatigue of caregivers

One person’s illness in the family upsets the family balance and changes the lifestyle of all family members [45]. Caregivers face problems with stress and emotional challenges, adjusting to new responsibilities, care and treatment issues, and declining QOL [46]. Due to the unpredictable nature of the disease, the activities of the families of people with MS are unplanned, and their spouses often feel pressured by the limited participation of their spouses in life. Chen et al. believe that if the QOL and efficiency of the family caregiver is improved, the effectiveness of the family caregiver in caring for the client will also be improved [47].

#### Information deficiency about MS

One of the problems raised by the participants in the research is the ignorance and little information of patients and people in the population about this disease, which has a significant impact on increasing the problems of patients. This finding differed from the results of a study conducted by Hung et al. They reported that at each stage of the disease, caregivers are provided with ongoing training tailored to the patients’ feelings and physical needs [48]. The results of a study done by de Seze et al. [49] showed that 86 patients (42.6%) and 70 patients (34.7%) respectively claimed to be well informed about the disease and its treatment. Adherence was significantly higher in well-informed patients. It seems that due to the increasing prevalence of people with MS in societies, public education about the symptoms and needs of these people is necessary so that society can take steps to properly support them. Therefore, it is necessary to design and implement intervention programs to increase public awareness of this disease at the society.

#### Family tensions

Most participants in the research stated that traumatic family factors were among the factors that started or aggravated their disease. Many studies have examined the relationship between stressful life events and the development of autoimmune diseases [50–52]. The results of a study carried out by Abdollahpour et al. [51] showed that periods of homelessness as well as divorce increase the risk of MS. However, marriage, the death of a loved one, and unemployment increase the risk of developing MS. Although most of the issues mentioned by the participants themselves as the cause or causes of the onset, exacerbation and aggravation are problems that many people have and that afflict any healthy person, it should be borne in mind that these patients, in addition to problems and disabilities due to illness are probably not able to deal with those problems due to lack of communication skills, problem solving and emotional intelligence. Therefore, it is essential that individual and group counseling and guidance services are helpful and complementary to medication in these cases.

#### Lack of social support

Some participants believed that lack of support or insufficient support from family and friends is an important and vital factor in their QOL. The results of a study done by Kristofferzon on the social support in chronic patients showed that about one-third of the samples reported low social support [53]. The results of a study conducted by Patel et al. [54] show that emotional support helps patients to rely on family support and feel that they are supporting the patient in these difficult and critical situations, and thus it will be easier for them to coping with this disease. In a study Krokavcova et al. found that more emotional, psychological, and psychosocial support from family and friends was directly related to improved mental health and MS patients’ response. This study emphasizes the power of the family in emotionally supporting patients, a support that should not be overlooked [55]. We can say that patients who have less social support are more prone to mental health problems, especially stress, and the severity of the disease is higher in these patients, and this can affect the coping and QOL of patients [56].

#### Fun and entertainment

The results showed that due to various restrictions on the physical and mental functioning of the patients, the participants were deprived of attendance in some places. The results of previous studies show that appropriate environmental conditions play an important role in the physical and mental health of patients with chronic diseases, because environmental characteristics and related factors affect fatigue, disease severity, physical activity, recreation and socializing is effective and can also affect the patients’ QOL [56]. Environmental conditions contribute to the development of many neurological and gastrointestinal disorders such as MS, Parkinson’s, etc. and have adverse effects on physical and mental health and well-being [57].

### Research Limitations

The results of this study are limited to the QOL barriers in patients with MS in Iranian culture, especially in Isfahan. Therefore, in order to benefit from these findings, we recommend further studies in different cultures and contexts. Due to the specific culture of Iran, especially its economic aspects, care must be taken in generalizing the results. The tone of voice and the feeling of security and calm that is induced in the interviewee, even in spite of the interviewer’s effort to be neutral in the interview session, are also among the limitations of this research. One of the positive points of this research was the use of the experiences of patients who were members of the MS Association along with the experiences of patients who did not benefit from the services of the MS Association.

## Conclusions

The results of this research showed that various factors have an inhibitory role in the QOL of patients with MS. These factors are divided into two categories: intrapersonal problems and environmental barriers. Thus, identifying these factors, in addition to raising our awareness, helps patients and their families, health care providers, as well as the society and government, to improve their QOL.

## Data Availability

The datasets used analyzed during the current study are not publicly available due the possibility that sharing interviews, which contain sensitive information about participants’ identities, may compromise participant anonymity, however, the quantitative data are available from the corresponding author on reasonable request.
